# Bidirectional, prospective associations between physical activity, fundamental motor skills, and physical fitness in preschool children

**DOI:** 10.1186/s12889-025-25871-7

**Published:** 2025-12-08

**Authors:** Elisabeth Straume Haugland, Eivind Aadland, Ada Kristine Ofrim Nilsen, Arne Ola Lervåg, Katrine Nyvoll Aadland

**Affiliations:** 1 Faculty of Education, Arts and Sports, Department of Sport, Food and Natural Sciences, Western Norway University of Applied Sciences, Campus Sogndal, Box 133, Sogndal, 6851 Norway; 2 Department of Education, University of Oslo, Oslo, Norway; 3 CREATE – Centre for Research on Equality in Education, University of Oslo, Oslo, Norway

**Keywords:** Fundamental movement skills, Motor competence, Health-related fitness, Kindergarten, Structural equation modelling, Accelerometry

## Abstract

**Background:**

Evidence regarding longitudinal associations between physical activity (PA), fundamental motor skills, and physical fitness in young children is scarce and mixed. This study aimed to investigate bidirectional associations between intensity-specific PA and fundamental motor skills and physical fitness in 3–4-year-old preschoolers over 18 months.

**Methods:**

We included 821 children (3.8 years, 53.5% boys) from the Active Learning Norwegian Preschool(er)s study who were measured at baseline and 18 months later. PA was measured with ActiGraph GT3X + accelerometers, fundamental motor skills were evaluated using 9 skills as indicators of locomotor skills, object control skills, and balance skills, and fitness was measured through tests of upper- and lower-body muscular strength (handgrip and standing long jump) and motor fitness (4 × 10 shuttle-run test). Structural equation modelling (SEM) with latent change-scores was used to investigate the bidirectional, prospective associations for PA with fundamental motor skills and physical fitness.

**Results:**

Vigorous PA (VPA) and total PA (TPA) were bidirectionally, and favourably, associated with locomotor skills and motor fitness. Moderate PA (MPA) and moderate-to-vigorous PA (MVPA) showed a positive association with balance skills, and MPA, VPA, MVPA, and TPA were prospectively and positively associated with object control skills, handgrip strength, and standing long jump, but not vice versa. Sedentary behaviour (SED) showed a bidirectional, negative association with locomotor skills, and a negative association with object control skills and all fitness measures.

**Conclusion:**

Although we found a few bidirectional associations for PA with locomotor skills and motor fitness, our results suggest that promoting MVPA in early childhood can have a positive impact on children’s development of fundamental motor skills and physical fitness.

**Supplementary Information:**

The online version contains supplementary material available at 10.1186/s12889-025-25871-7.

## Background

The early years are a critical period to establish sufficient levels of PA [1] and concomitant developmental outcomes such as fundamental motor skills (FMS) and physical fitness (FIT) [2, 3]. Satisfactory levels of FMS (e.g., locomotor skills, object control skills, and balance skills) and FIT (e.g., muscular fitness and motor fitness) during childhood may provide a foundation for PA participation [4, 5] and the development of more complex skills [2, 6], and is likely associated with better health later in life [7]. The relationship between PA, FMS, and FIT are conceptualized to be bidirectional and differ through a child`s developmental stages. In early childhood (2–5 years), it is suggested that PA drives FMS development, while FMS may underpin and drive PA engagement with increasing age as children become more motor competent [2]. While the longitudinal relationships between PA, FMS and FIT have been explored in children [8], evidence on the relationships between PA and FMS, and between PA and FIT is limited in young children. More knowledge of the direction of these relationships in early childhood is needed to inform intervention development, policy, and practice [9–12].

Among the relatively few longitudinal studies in preschool children, some have shown no associations between PA at baseline and FMS at follow-up [13–16], while others have found PA to positively predict future FMS, especially locomotor skills [17–19]. One previous study in preschoolers also found PA to positively predict change in object control and balance skills [19]. At the same time, sedentary time (SED) has been found to negatively predict change in locomotor skills [19] or to be unrelated to future FMS levels [20]. Regarding the opposite pathway, studies investigating FMS as exposure for future PA levels in preschoolers suggest that total FMS [21–23] or locomotor skills, but not object control skills [24–26], positively predict future light PA (LPA) and/or MVPA, while locomotor skills have been found to be negatively associated with future SED [24, 26]. Importantly, several of these studies are limited by the lack of adjustment for baseline levels of the outcome [17, 20–22, 25, 26]. The few studies that have investigated bidirectional associations between PA and FMS have found no evidence for FMS to predict change in PA of any intensity [15, 16, 19]. While a few studies have applied longer follow-ups (i.e., 2–5 years) [13, 15, 19, 26], the majority of the aforementioned studies have short follow-up periods of approximately one year, and several studies are limited by including only a total FMS score or scores for locomotor or object control skills, and not balance skills [13, 16].

Studies targeting preschool children suggest that engaging in more PA of higher intensities predicts improved change in upper-body strength [27, 28], lower-body strength [27–30], and motor fitness [27, 28, 30], while others have found no prospective associations between PA and these measures [31]. Although some studies have provided prospective evidence for an association, sample sizes are relatively small (*n* = 138–262) [27–30], possibly leading to variability in estimates. Only one study to date has investigated the longitudinal bidirectional associations between PA and FIT in younger children from 4 to 9 years of age, showing no direct associations of the movement behaviours at 4 years with physical fitness at 9 years [31].

On this background, there is a need for comprehensive large-scale research investigating the relationship between PA, FMS, and FIT using robust methods. Applying SEM, specifically latent growth models, allows for estimating complex models including multiple variables [32]. Therefore, this study aims to investigate bidirectional, prospective pathways for PA with FMS and FIT using SEM in 3–4-year-old children over 18 months.

## Methods

### Study design and participants

This is a prospective longitudinal study where children were assessed at baseline and 18 months later. The study used data from the Active Learning Norwegian Preschool(er)s study (ACTNOW) conducted in Sogn and Sunnfjord regions in Norway 2019–2022. The ACTNOW protocol has previously been published [33]. ACTNOW is a two-arm cluster randomized controlled cluster trial (RCT) randomized at the preschool level. Preschools in the selected regions having ≥ six 3–4-year-old children enrolled were invited to participate, and all children aged 3–5 years at baseline within the participating preschools were invited to take part in the study. In the present study, the total sample irrespective of group allocation, is included for prospective analyses.

### Ethics statement

Children’s parent(s) or guardian(s) were provided oral and written information about the ACTNOW study and provided written consent before testing. All testing was conducted in preschools, in safe and familiar surroundings. Children were informed about the study at their level of understanding. The Norwegian Center for Research Data (NSD, ref. nr. 248220) and the institutional ethics committee at Western Norway University of Applied Sciences approved the ACTNOW study. Procedures and methods adhere to the ethical guidelines outlined by the World Medical Association`s Declaration of Helsinki and its subsequent revisions [34]. The study was registered on clinicaltrials.gov (https://clinicaltrials.gov/ct2/show/NCT04048967?term=actnow&rank=1) on August 7th, 2019 (ID: NCT04048967).

### Measurements

#### Anthropometry and demography

Parental education level (SES, highest of mother/father) was reported by parent(s) or guardian(s), and responses were categorized into (a) upper or lower secondary school, (b) university < 4 years and (c) university ≥ 4 years. Children’s weight and height were measured according to the Assessing FITness in PREschoolers battery (PREFIT) battery [35]. Weight was measured to the nearest 0.1 kg using an electronic scale (Seca 899, SECA GmbH, Hamburg, Germany), and height was measured to the nearest 0.1 cm using a transportable Seca 217 (SECA GmbH, Hamburg, Germany). Body mass index (BMI) (kg/m^2^) was calculated. Age- and sex-specific cut points by Cole et al. [36] were used to categorize children as normal weight, overweight, or obese.

#### Physical activity

PA was assessed using the ActiGraph GT3X + accelerometer (ActiGraph, LLC, Pensacola, Florida, USA) [37], which is a widely used and validated method [38, 39]. Children were asked to wear the accelerometer on the right hip 24 h/day (except during water-based activities) for seven consecutive days at both measurement timepoints. Sampling rate was set to 30 Hz, and data were analyzed using 1-s epochs [40] using a custom-made script in MATLAB (MathWorks, Massachusetts, USA). Non-wear time was defined as consecutive periods of ≥ 20 min of zero counts [41]. Children had to achieve ≥ 8 h/day of wear time for ≥ 3 valid weekdays and ≥ 1 valid weekend day to be included [42]. PA was reported as total PA (counts per minute [cpm]) and minutes/day spent in intensity-specific PA and SED as determined by Evenson et al. [43] (SED (≤ 100 cpm), LPA (101–2295 cpm), MPA (2296–4011), VPA (≥ 4012 cpm), and MVPA (min/day) (≥ 2296 cpm)). To control for wear time, the percentage of time spent in different PA intensities was used in association analyses.

#### Fundamental motor skills

To evaluate FMS, a modified test battery guided by the Test of Gross Motor Development 3 (TGMD-3) [44] and the Preschooler Gross Motor Quality Scale (PGMQS) [45] was used. It was compiled of locomotor- (running, jumping, hopping) and object control (overhand throw, catch, kick) skills from the TGMD-3 and balance skills (single leg standing, walking on line forward and backward) from PGMQS. The use of a modified assessment tool allowed for including all three FMS domains, reducing the participant and researcher burden, in addition to including skills more relevant for a Norwegian activity context. Relevant items that was deemed representative and feasible for locomotion and object control skills were chosen. The structural validity of the test battery is acceptable despite significant commonality between locomotor and object control skills [46]. FMS was tested and scored according to existing protocols for TGMD-3 (locomotor- and object control items) and PGMQS (balance items). One instructor explained and demonstrated one skill at a time during testing, while a separate assessor scored the children’s performance. Children were scored quantitatively based on the evaluation of whether or not the child demonstrated specific process criteria (either “0” or “1”) [44, 45]. Each child had one test attempt per skill before performing each skill twice in a standardized order. Scores from both trials were summed, scoring 0–2 points per criteria. Scores for each domain were summed, providing a score between 0 (lower skill level) and 22 for object control skills or 24 for locomotion and balance skills. Inter-rater reliability (based on video-scoring of 22 children performed a priori to the ACTNOW study) was 0.76–0.85 across domains [46]. Due to some variation between assessors, FMS scores were adjusted for assessors in the statistical analysis. To correct for differences between raters, we calculated mean scores for each rater across all children included in an interrater reliability study, and adjusted all scores in the larger study for rater by correcting for the difference with a reference rater (i.e., the most experienced rater).

#### Physical fitness

Motor fitness and muscular fitness were assessed with the PREFIT battery [35], which is designed for and has shown good reliability in preschool populations [47, 48]. We used the 4 × 10 m shuttle run test to assess motor fitness, which assesses speed and agility. Children were asked to run as fast as possible back and forth between cones placed 10 m apart. They performed the task twice, and the best result were reported in analyses (measured in seconds). The handgrip strength test for upper-body strength was tested twice for each hand with a hand dynamometer (TKK 5001 Grip A, analogue model 577, Takey, Tokio) with a standardized grip span of 4.0 cm [49, 50]. Results were reported in kg and the highest of four scores (two left hand, two right hand) were used in the analyses. Assessment of lower-body strength was performed with the standing long jump test. Children were instructed to jump (two-footed take-off and landing) as far as possible from a standing position after one test jump. Children’s performance was scored twice. The best of two valid performances was used, reported in cm.

### Statistical analyses

**Fig. 1:**
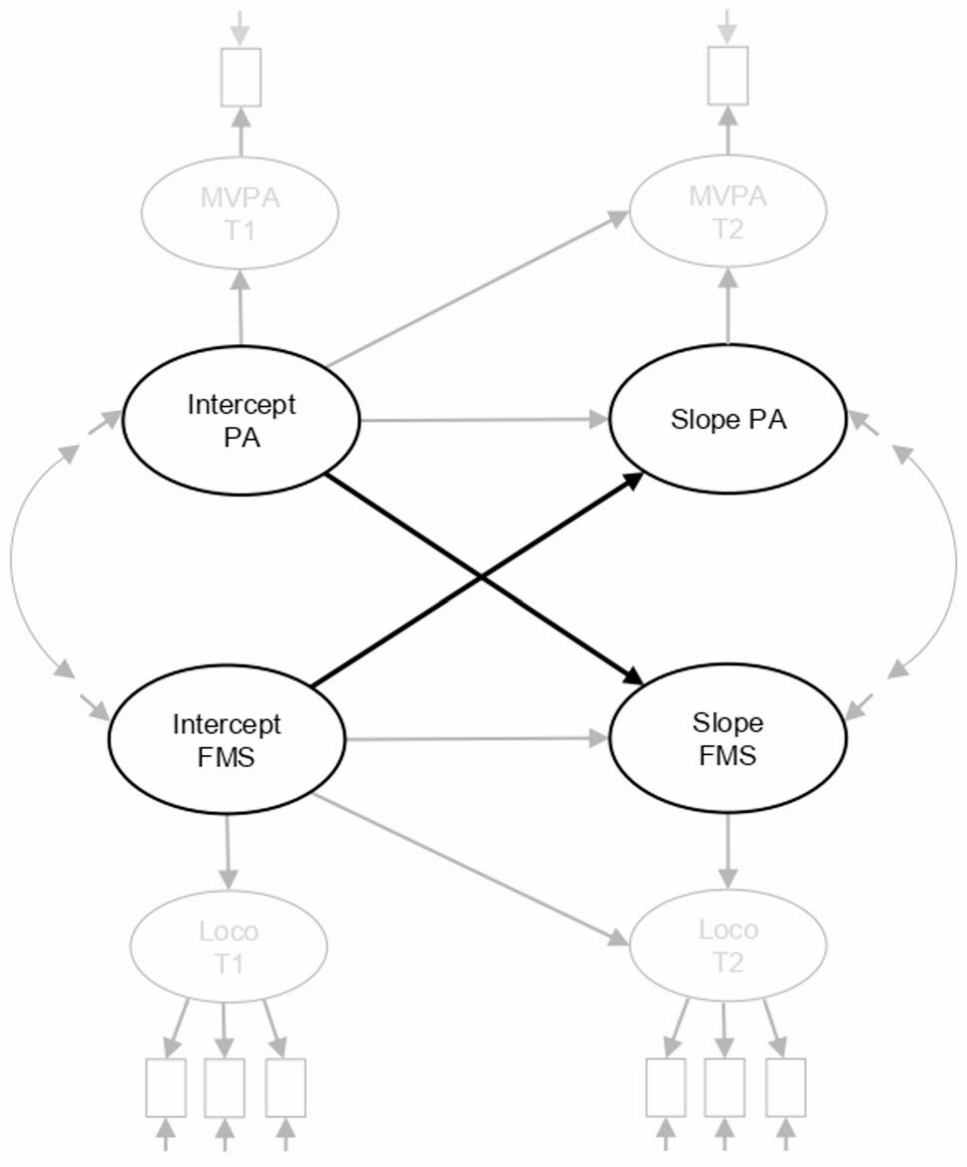
Example model of the latent growth model used in current analyses, including intercept PA (baseline PA), slope PA (change PA), intercept FMS (baseline FMS), and slope FMS (change FMS). Bold arrows indicate estimated paths showing prospective association for baseline PA with change in FMS/FIT, or vice versa. Remaining parts indicate underlying parts of the model. PA = Physical Activity, FMS = Fundamental Motor Skills, MVPA = Moderate-to-vigorous physical activity, Loco = locomotor skills

Children’s characteristics were reported as means and standard deviations (SD) or frequencies. Descriptive analyses were performed using IBM SPSS v. 29 (IBM SPSS Statistics for Windows, Armonk, NY; IBM Corp., USA). Confirmatory factor analyses (CFA) and growth/latent change-score models were performed using Mplus, version 8 (Muthén and Muthén, Los Angeles, USA). We used latent growth models [51] within the SEM framework to calculate latent change scores that were used to determine bidirectional associations for PA with FMS and FIT. In these models, we estimated an ‘intercept’ equaling the scores at the first time point, and a ‘slope’ equaling the change between the first and the second time point (18-month follow-up). Figure 1 shows the model paths, exemplified with latent change scores of PA and FMS, used to estimate the intercepts and slopes. We regressed the slopes on both its own intercept (i.e., adjusting for baseline) and on the intercept of the other construct (i.e., showing the prospective association for baseline PA with change in FMS/FIT, or vice versa). These models allow for correcting for measurement error because they use latent variables representing changes over time and thus separate true differences in change over time from random measurement error [32].

To account for the preschool cluster, the complex method with the robust maximum likelihood (MLR) estimator, which allows for maximizing available data, was used in growth models and measurement invariance analyses. Measurement invariance testing was performed for the three latent factors, locomotor, object control, and balance, to evaluate whether the temporal change of the constructs was due to true change or to changes in the measurement of the construct over time [32]. Measurement invariance was also tested across gender.

Growth modelling was performed separately for each FMS domain and FIT measure, with each PA intensity or SED (36 models). We used standardized beta-coefficients (β) to evaluate the significance and direction of associations. All models were adjusted for sex, age, BMI, parents’ education, and group allocation since we included children from both the intervention and control groups. We applied the Wald test of parameter constraints to test for differences in associations between boys and girls.

It is recognized that PA measurements typically have modest reliability as behaviour vary over time. This error should be taken into account to avoid misleading conclusions being drawn [52–54]. We corrected PA intensity variables for measurement error by adjusting their variances [32] (based on the full sample) by week-by-week reliability estimates for accelerometer-determined PA from Aadland et al. [42]. Measurement error in FMS items were estimated based on internal consistency estimates (McDonald’s Omega) of the items’ underlying criteria and the variance of each variable in question from the complete sample.

Due to a large sample size, multiple indices were used to evaluate the model fit in invariance testing and growth models; a Comparative Fit Index (CFI) where values greater than 0.90 were indicative of “good fit” and values greater than 0.95 were considered “excellent”, a Root Mean Squared Error of Approximation (RMSEA) ≤ 0.06, and a Standardized Root Mean Squared Residual (SRMR) ≤ 0.08 [32, 55]. Statistical significance was set to *p* ≤ 0.05 in all models.

## Results

### Sample characteristics

A total of 46 preschools accepted the invitation to participate in the study (82%), encompassing 1533 children. Out of the invited participants, 1265 had consent to participate (82%). Starting school prior to follow-up measurements were the main reason for being excluded from analysis (*n* = 443). Included children (*n* = 821, Table 1) provided valid data on either exposure or outcome variables (FMS and/or FIT, or PA). Two children provided data on only FMS or FIT, thus resulting in 820 participants in FMS analyses, and 820 participants in FIT analyses.

**Table 1:** Descriptive data of included children reported as means (SD) or frequencies (%)

	*n*	Baseline	*n*	Follow-up
*Demographics*				
Sex (% boys)	821	53.5		-
Age (years)	820	3.8 (0.6)	781	5.3 (0.6)
Parents’ education level (%)	751			
≤ Upper secondary school		26.5		-
University < 4 years		28.2		-
University ≥ 4 years+		45.3		-
*Anthropometry*				
BMI (kg/m^2^)	775	16.4 (1.4)	769	16.1 (1.6)
Normal weight (%)		84.0		83.2
Overweight (%)		13.4		13.4
Obese (%)		2.6		3.4
*Fundamental motor skills*				
Locomotor skill score	712	8.9 (3.8)	746	13.7 (3.9)
Object control skill score	745	6.6 (3.0)	753	9.9 (3.9)
Balance skill score	705	7.6 (5.2)	755	12.8 (4.9)
*Physical fitness*				
Handgrip strength (kg)	745	7.0 (2.1)	768	9.8 (2.4)
Standing long jump (cm)	677	67.1 (23.2)	709	95.2 (18.5)
Motor fitness (sec)	712	19.6 (2.8)	746	16.6 (2.1)
*Physical activity full day*	666		588	
Wear time (min/day)		759.1 (68.6)		770.6 (71.7)
Total PA (cpm)		657.4 (140.7)		745.5 (177.0)
SED (min/day)		535.1 (63.2)		540.7 (68.4)
LPA (min/day)		152.5 (21.2)		148.6 (20.5)
MPA (min/day)		36.7 (7.3)		40.1 (7.6)
VPA (min/day)		34.8 (9.5)		41.2 (11.2)
MVPA (min/day)		71.5 (16.0)		81.3 (17.2)
PA guideline* (%)		77.3		87.9

Weight status according to Cole et al. [36]. SES defined as highest educational level of the child`s mother or father

*SD* Standard Deviation; *BMI * Body Mass Index

*PA guidelines defined by the Norwegian health directorate [56] and WHO [57], 180 min/day in any intensity, of which 60 min/day in MVPA. PA intensity categories defined according to Evenson et al. [43]

FMS: Fundamental motor skill, sum score between 0 (lower skill level) and 22 (for object control skills) or 24 (for locomotion and balance skills)

### Invariance testing

The latent factors for object control and balance showed scalar invariance over time (object control *p* = 0.093; balance *p* = 0.779) indicating that the different items load equally at both timepoints. The locomotor domain had partial scalar invariance (*p* = 0.089) where the intercept of the ‘jump’ indicator was invariant across time (and therefore freely estimated). We therefore performed a sensitivity analysis for the jump indicator (same analysis as for the latent factors), which provided similar results as the original analyses with the latent locomotor factor (Additional file 1: Table S1). As we did not have measurement invariance across gender, the nine individual indicators were used in these analyses.

### Bidirectional associations between physical activity and fundamental motor skills

All PA intensities at baseline positively predicted change in locomotor skills (*p* < 0.001–0.019) (Table 2). All PA intensities except LPA (*p* = 0.066) positively predicted change in object control skills (*p* = 0.001–0.033), while baseline MPA and MVPA positively predicted change in balance skills (*p* = 0.001–0.010). SED predicted a negative change in locomotor skills (*p* = 0.001) and object control skills (*p* = 0.007) but was unrelated to change in balance skills (*p* = 0.138).

**Table 2:** Standardized coefficients for paths between physical activity and motor skills, and goodness of fit indices

	Intensity	PA ➔ FMS	FMS ➔ PA	Chi-square (df)	CFI	RMSEA (90% CI)	SRMR
Locomotor skills	TPA	**0.305 (< 0.001)**	**0.314 (0.013)**	76.916 (39)	0.965	0.034 (0.022–0.045)	0.028
	MVPA	**0.310 (< 0.001)**	**0.255 (0.046)**	76.088 (39)	0.968	0.034 (0.022–0.045)	0.029
	VPA	**0.274 (< 0.001)**	**0.279 (0.041)**	77.807 (39)	0.966	0.035 (0.023–0.046)	0.028
	MPA	**0.313 (< 0.001)**	0.161 (0.143)	73.104 (39)	0.970	0.033 (0.021–0.044)	0.029
	LPA	**0.169 (0.019)**	0.085 (0.289)	71.448 (39)	0.969	0.032 (0.020–0.043)	0.027
	SED	**−0.243 (0.001)**	**−0.209 (0.032)**	73.143 (39)	0.968	0.033 (0.021–0.044)	0.028
Object control skills	TPA	**0.225 (0.033)**	−0.147 (0.526)	106.266 (41)	0.907	0.044 (0.034–0.054)	0.033
	MVPA	**0.244 (0.003)**	−0.030 (0.880)	107.537 (41)	0.916	0.044 (0.034–0.055)	0.034
	VPA	**0.195 (0.030)**	−0.150 (0.459)	106.023 (41)	0.916	0.044 (0.034–0.054)	0.033
	MPA	**0.270 (0.001)**	0.125 (0.473)	109.208 (41)	0.916	0.045 (0.035–0.055)	0.034
	LPA	0.105 (0.066)	0.141 (0.179)	105.911 (41)	0.918	0.044 (0.034–0.054)	0.034
	SED	**−0.180 (0.007)**	−0.139 (0.366)	107.410 (41)	0.913	0.044 (0.034–0.055)	0.034
Balance skills	TPA	0.103 (0.121)	−0.092 (0.539)	43.067 (41)	0.998	0.008 (0.000–0.026)	0.017
	MVPA	**0.136 (0.010)**	0.051 (0.697)	39.779 (41)	1	0.000 (0.000–0.023)	0.017
	VPA	0.103 (0.077)	−0.030 (0.804)	41.501 (41)	1	0.004 (0.000–0.024)	0.017
	MPA	**0.163 (0.001)**	0.157 (0.230)	37.902 (41)	1	0.000 (0.000–0.021)	0.017
	LPA	0.029 (0.602)	0.071 (0.411)	38.192 (41)	1	0.000 (0.000–0.021)	0.017
	SED	−0.082 (0.138)	−0.085 (0.445)	38.542 (41)	1	0.000 (0.000–0.022)	0.017

Models are adjusted for sex, age, BMI, parents’ education level, FMS assessor, and group allocation

*TPA* Total physical activity, *MVPA* Moderate-to-vigorous physical activity, *VPA* Vigorous physical activity, *MPA* Moderate physical activity, *LPA* Light physical activity, *SED* Sedentary behaviour, *CFI* Comparative Fit Index, *RMSEA* Root Mean Square Error of Approximation, *SRMR* Standardized Root Mean Squared Residual

Estimate (*p*-value) for paths. PA-FMS; FMS-PA

Significant associations are marked in bold. *N* = 820

Locomotor skills at baseline predicted a positive change in TPA, MVPA, and VPA (*p* = 0.013–0.046) and was negatively associated with change in SED (*p* = 0.032). Object control skills and balance skills at baseline did not predict change in PA of any intensity or SED (*p* = 0.179–0.880). Associations did not differ between girls and boys (p for interactions = 0.072–0.997, results not shown).

### Bidirectional associations between physical activity and physical fitness

All PA intensities except LPA (*p* = 0.119) positively predicted change in handgrip strength (*p* = 0.007–0.033) and standing long jump (*p* < 0.001) (Table 3). All PA intensities favourably predicted change in motor fitness (*p* < 0.001–0.007), whereas SED negatively predicted change in all fitness measures (*p* < 0.001–0.034). For the pathway from FIT to PA, motor fitness predicted favourable changes in TPA and VPA (*p* = 0.003–0.018). Prediction of pathways did not differ between boys and girls (*p* = 0.105–0.951, results not shown) except for one pathway where standing long jump negatively predicted LPA in boys (*p* = 0.041) but not in girls.

**Table 3:** Standardized coefficients for paths between physical activity and fitness measures

	Intensity	PA ➔ FIT	FIT ➔ PA
Handgrip strength	TPA	**0.187 (0.010)**	0.052 (0.575)
	MVPA	**0.164 (0.013)**	0.034 (0.657)
	VPA	**0.171 (0.007)**	0.086 (0.236)
	MPA	**0.134 (0.033)**	−0.039 (0.620)
	LPA	0.072 (0.119)	−0.094 (0.207)
	SED	**−0.121 (0.034)**	0.037 (0.645)
Standing long jump	TPA	**0.249 (< 0.001)**	0.082 (0.337)
	MVPA	**0.213 (< 0.001)**	0.036 (0.605)
	VPA	**0.240 (< 0.001)**	0.114 (0.140)
	MPA	**0.150 (< 0.001)**	−0.072 (0.213)
	LPA	0.018 (0.629)	−0.079 (0.114)
	SED	**−0.102 (0.003)**	0.015 (0.787)
Motor fitness	TPA	**−0.198 (< 0.001)**	**−0.232 (0.018)**
	MVPA	**−0.184 (< 0.001)**	−0.146 (0.143)
	VPA	**−0.161 (< 0.001)**	**−0.247 (0.003)**
	MPA	**−0.188 (< 0.001)**	0.039 (0.712)
	LPA	**−0.106 (0.007)**	−0.002 (0.978)
	SED	**0.151 (< 0.001)**	0.096 (0.272)

Models adjusted for sex, age, BMI, parents’ education level, FMS assessor, and group allocation

*TPA* total physical activity, *MVPA* Moderate-to-vigorous physical activity, *VPA* Vigorous physical activity, *MPA* Moderate physical activity, *LPA* Light physical activity, *SED* Sedentary behaviour

Estimate (*p*-value) for paths PA-FIT; FIT-PA using observed fitness-scores (saturated model, i.e., no model fit indices)

Significant associations are marked in bold. *N* = 820. Lower motor fitness estimates indicate better performance

To improve understanding of the associations between PA and FMS and FIT we have presented results in which PA measurement error has been minimized. Models with (Additional file 2–3: Table S2 and S3) and without (Tables 2 and 3) PA measurement error were generally similar across FMS domains but varied for FIT measures where a few pathways from all FIT measures to high intensity PA became non-significant when PA measurement error was removed. We performed sensitivity analyses including only the control group, for which results were similar to the main analyses for FMS (Additional file 4: Table S4) and FIT (Additional file 5: Table S5), except for handgrip strength for which associations were non-significant (*p* ≥ 0.142).

## Discussion

The present study examined bidirectional associations between intensity-specific PA, domain-specific FMS, and different FIT measures in a large sample of preschoolers. We found that VPA presented a favourable and bidirectional association with locomotor skills and motor fitness. Moreover, MVPA levels positively predicted change in object control skills, balance skills, handgrip strength, and standing long jump (outcome), but not vice versa. While only locomotor skills negatively predicted change in SED, baseline SED negatively predicted change in all FMS and FIT measures except balance skills. Results were similar for boys and girls. Our results suggest that PA is a stronger predictor of development in FMS and FIT in early childhood than vice versa, thus supporting the conceptual model by Stodden and colleagues [2].

The current study shows that PA predicted change in FMS, contributing important knowledge to current evidence on the longitudinal association between PA and FMS in early childhood. Notably, only a few longitudinal studies of preschool children have been included in previous reviews [8, 11, 58], underlining the need for more high-quality research in this population. Although several previous studies in preschool children show no longitudinal associations between PA (exposure) and FMS (outcome) [13, 15, 16], our results concur with Nilsen et al. [19], who found that MVPA (exposure) positively predicted change in locomotor, object control, and balance skills (outcomes) and that SED negatively predicted change in these outcomes. Other studies in preschool children have also shown MVPA to positively predict future locomotor skills but not object control skills [17, 18] or balance skills [14]. Similar to our findings, a few studies in preschool children have found that locomotor skills, but not object control skills (exposure), positively predicted future MVPA (outcome) [24–26]. Studies have suggested that associations between PA and FMS are weak in younger children and is expected to strengthen with increasing age [59]. This is in line with our results showing significant, but weak, associations between PA and the FMS domains. Thus, based on the cross-sectional study by Barnett and colleagues, we hypothesize associations would strengthen over time, but how maturation affect directionality of these associations needs to be addressed in future longitudinal studies. As children tend to learn object control skills at a later stage than locomotor skills [60], these skills may not yet have emerged in their movement trajectories at the ages of 3–5 years, in line with Stoddens’ model [2], and might contribute to explain our weaker, and only unidirectional associations between PA and object control skills. While some studies have found object control skills to predict future PA in older children [61, 62], recent evidence has shown indeterminate evidence for the relationship between object control skills and PA in youth [8]. As in the current study, PA is usually interpreted and discussed in terms of intensity as opposed to by type or quality of movements [63]. Although object control skills may be related to MVPA through a range of games and activities, upper body movements typical for object control skills (i.e., throwing, catching) and the intensity of such intermittent movements characterizing ball games may not be captured optimally by hip-worn accelerometers [64].

Our results show that MVPA predicted a change in balance skills but not vice versa. The three FMS domains may have different associations with PA intensities, which makes it likely that they would represent various types of activities [9]. However, balance skills are considered an underlying ability and prerequisite necessary to master any gross motor activity [60, 65]. Balance skills may be understood as a basic characteristic and, therefore, something different from the more tangible object control or locomotor skills. Further, activities involving particular balance skills might not be captured as accurately by accelerometry in comparison to object control and locomotor skills and might also potentially be more difficult to measure precisely due to its less specific characteristics, contributing as a possible explanation to our few, and weaker, associations for balance skills.

We found that PA generally predicted change in FIT but not vice versa. While the only previous study that has explored the bidirectional association between PA and FIT found no significant associations for motor fitness or upper- or lower-body muscle strength [31], we found that MVPA positively predicted change in all FIT measures, being strongest for standing long jump. Although Migueles et al. [31] used the same test battery as the current study, differences in follow-up (5 years), statistical modelling and sample size (*n* = 201) limit direct comparison. Other studies in young children using objective measures for PA aligns with our findings that MVPA favourably predicts change in motor fitness [28, 30], standing long jump [27–30], and handgrip strength [27, 28]. However, as these studies only examined the unidirectional association, more research is needed to explore the bidirectional nature of this relationship. The current study found few bidirectional associations, thus, supporting the hypothesis that PA, particularly in early childhood, might drive development of FIT and FMS [2]. Young children need to engage in PA to develop skills, whilst the need for certain skill levels may be more important later in childhood when participating in sports and physical education in school.

Current inconsistent evidence may be a result of some studies not measuring domain-specific FMS [13, 16, 22], thus not capturing the nuances of these relationships. In addition, most studies have low to moderate sample sizes (9 studies *n* ≤ 256 [13–15, 17–19, 21, 24, 25]; 3 studies *n* = 441–555 [16, 22, 26]), and applied a variety of FMS measurements (i.e., TGMD, The Movement Assessment Battery for Children, the Körperkoordinationtest für Kinder, the Zurich Neuromotor Assessment), which likely cause variability across studies. The few studies investigating bidirectional associations between PA and FMS have not found FMS to predict future PA [15, 16, 19], and only Nilsen et al. [19] found (MV)PA to predict future FMS. Although the current study found some bidirectional associations, there were a higher number of, and stronger associations for PA to FMS than vice versa. Hence, participating in varied PA in early childhood is likely more important for future FMS, than the actual FMS level is for future PA participation, which aligns with Stodden et al.’s hypothesis [2]. Although the same FMS assessment was used in Nilsen et al. [19] as in the current study, differences in sample size and analytical approach may explain the minor differences in results. Direct comparison of associations from the current study with previous literature is further complicated by variety in baseline age, follow-up duration, different data reduction methods for accelerometry (i.e., e-poch length and intensity cut-points), and analytical approaches. Although different types of analyses may contribute to confusion in interpreting and comparing results, similar prospective findings for FMS and FIT in young children have been found across multiple linear regression analysis [18, 19, 24, 26, 28, 30], multivariate pattern analysis [29], compositional data analysis [27], isotemporal substitution analysis [30] and SEM analysis (present study). The present study extends current findings by including balance and FIT measures and by choosing an analytical approach that handles complex path models and measurement error, as opposed to common regression analysis.

### Strengths and limitations

This study’s main strengths are the analytical approach using SEM and the inclusion of a large study sample, allowing for simultaneously estimating latent variables and the causality between them, with correction for measurement error. Although no gold standard exists for measuring FMS [66], the inclusion of skills from standardized assessments (TGMD-3 and PGMQS) is considered a strength; however, we used a modified assessment battery for FMS, limiting comparability with the full versions and total scores from TGMD-3 and PGMQS [46]. We regard the inclusion of balance skills as a strength in the current study as this FMS domain is usually overlooked, although equally recognized as an FMS domain [67]. Although we did not include measures of cardiorespiratory fitness due to limited space in preschools (e.g., the 20 m shuttle run test from PREFIT requires a 25 m running area), we broadly captured different aspects of child development by including both FMS and FIT measures.

The increasing use of accelerometry for measuring PA allows for easier comparison across studies [68]; however, several challenges remain prominent. Accelerometry is limited by not accurately capturing the type of PA, and that it underestimates intensity in object control skills [64]. Accounting for measurement error in PA and FMS is a strength of the present study. FIT measurements are product-oriented tests and are therefore regarded as less prone to measurement error compared to assessing FMS, which is process-oriented in the current study. Although fairly similar results were found for models with and without correction for measurement error, coefficients in the models adjusted for measurement error were generally stronger when PA was modelled as exposure, whereas coefficients were either weaker or similar when FMS or FIT was modelled as exposure. We argue that our results, despite minor differences between models, obtained greater precision in associations by correcting for measurement error compared to applying traditional analyses (e.g., multiple linear regression). Sensitivity analyses performed in the control group showed similar results for all measures except handgrip strength, which showed no significant associations contrary to the primary analyses. However, we chose to present results that included both groups with adjustment for group allocation to maintain a higher power in our estimates. We have no reasonable explanation for these differences, and results for handgrip strength should, therefore, be interpreted cautiously. We did not explore the prospective relationship between FMS and FIT, as FMS and FIT are strongly correlated [69, 70].

Some research has found different developmental trajectories between genders during early childhood regarding FMS [71] and PA [72]. In line with previous studies not finding sex to moderate the relationship between PA and FMS [16] or PA and FIT [30], the current study found similar associations for girls and boys. The current sample’s narrow age span (within what Stodden et al. characterize as early childhood) limits our ability to draw conclusions concerning the reciprocal association between the analysed variables from early to middle childhood. We urge future studies to investigate bidirectional associations between PA and FMS and FIT with a longer follow-up duration with multiple measurements stretching from preschool to school age. This will allow for exploring the hypothesized relationship that strengthens with age [2], in addition to greater developmental nuances across time. While the present study results are comparable to other samples of healthy preschool children without known disabilities, generalisation should be made with caution as this study was conducted in a rural area in Western Norway and PA practices may differ across cultures and countries. Moreover, parental educational level is somewhat higher in our study than among the general adult Norwegian population.

## Conclusions

We used latent change models to determine the bidirectional, prospective associations between PA (and SED) and FMS and FIT in a large sample of preschool children. VPA presented a favourable bidirectional relationship with locomotor skills and motor fitness, whereas MVPA (exposure) demonstrated a positive unidirectional relationship with object control skills, balance skills, handgrip strength, and standing long jump (outcome). SED was adversely associated with all measures except balance skills. These results contribute to improve our understanding of children’s developmental trajectories and suggest that researchers and practitioners should promote MVPA in early childhood to positively affect children’s development of FMS and FIT.

## Supplementary Information

Supplementary Material 1.

## Data Availability

The datasets used and analyzed in this study are available from the corresponding author upon reasonable request.

## Abbreviations

BMI: Body mass index
CFI: Comparative Fit Index
cpm: Counts per minute
FMS: Fundamental motor skills
LPA: Light physical activity
min: Minutes
MPA: Moderate physical activity
MVPA: Moderate-to-vigorous physical activity
PA: Physical activity
RMSEA: Root Mean Squared Error of Approximation
SD: Standard deviation
SED: Sedentary time
SEM: Structural equation modelling
SES: Socioeconomic status
SRMR: Standardized Root Mean Square Residual
VPA: Vigorous physical activity

## Declarations

### Trial registration

clinicaltrials.gov, identifier NCT04048967, registered August 7, 2019, https://clinicaltrials.gov/ct2/show/NCT04048967?term=actnow&rank=1.
