# Supplementary material for: Bidirectional, prospective associations between physical activity, fundamental motor skills, and physical fitness in preschool children
Source: BMC Public Health. 2025 Dec 8;26:166. doi: 10.1186/s12889-025-25871-7 (PMC12797888; doi:10.1186/s12889-025-25871-7)
Supplement: Supplementary file 1 — Supplementary Material 1. [file 12889_2025_25871_MOESM1_ESM.pdf]

Additional file 1

Table S1. Standardized coefficients from sensitivity analyses of the ‘jump’ item.

	Intensity		Contains ME		
Jump			**PA→FMS**		**FMS→PA**
	TPA		**0.181 (<0.001)**		**0.164 (0.001)**
	MVPA		**0.177 (<0.001)**		**0.140 (0.003)**
	VPA		**0.178 (<0.001)**		**0.154 (0.002)**
	MPA		**0.154 (<0.001)**		**0.090 (0.043)**
	LPA		0.071 (0.108)		0.063 (0.166)
	SED		**-0.130 (0.002)**		**-0.118 (0.011)**
			**Corrected for ME (item and PA)**		
	TPA		**0.294 (0.005)**		**0.363 (0.049)**
	MVPA		**0.257 (0.003)**		**0.290 (0.045)**
	VPA		**0.240 (0.011)**		0.315 (0.063)
	MPA		**0.248 (0.005)**		0.184 (0.124)
	LPA		0.126 (0.141)		0.136 (0.167)
	SED		**-0.194 (0.022)**		**-0.255 (0.024)**

Estimate (p-value) for paths PA-FMS; FMS-PA. ME=Measurement error, TPA=total physical activity, MVPA=Moderate-to-vigorous physical activity, VPA=Vigorous physical activity, MPA=Moderate physical activity, LPA=Light physical activity, SED=Sedentary behavior. Models adjusted for sex, age, BMI, parents’ education level, FMS test person, and group. Significant associations are marked in bold.

Additional file 2

Table S2. Standardized coefficients for paths between physical activity and fundamental motor skills, and goodness of fit indices for the longitudinal models not corrected for measurement error.

	**Intensity**		**Contains PA ME**				**Model fit**			
			**PA→FMS, est (p)**		**FMS→PA, est (p)**		**Chi-square (df)**	**CFI**	**RMSEA (90% CI)**	**SRMR**
**Locomotor skills**										
	TPA		**0.229 (<0.001)**		**0.322 (<0.001)**		76.916 (39)	0.965	0.034 (0.022-0.045)	0.028
	MVPA		**0.249 (<0.001)**		**0.281 (<0.001)**		76.088 (39)	0.968	0.034 (0.022-0.045)	0.029
	VPA		**0.210 (<0.001)**		**0.319 (<0.001)**		77.808 (39)	0.966	0.035 (0.023-0.046)	0.028
	MPA		**0.264 (<0.001)**		**0.176 (0.022)**		73.104 (39)	0.970	0.033 (0.021-0.044)	0.029
	LPA		**0.155 (0.018)**		0.078 (0.239)		71.448 (39)	0.969	0.032 (0.020-0.043)	0.027
	SED		**-0.212 (0.001)**		**-0.200 (0.005)**		73.143 (39)	0.968	0.033 (0.021-0.044)	0.028
**Object control skills**										
	TPA		**0.156 (0.036)**		0.080 (0.487)		106.267 (41)	0.907	0.044 (0.034-0.054)	0.033
	MVPA		**0.197 (0.004)**		0.086 (0.485)		107.536 (41)	0.916	0.044 (0.034-0.055)	0.034
	VPA		**0.151 (0.032)**		0.040 (0.722)		106.023 (41)	0.916	0.044 (0.034-0.054)	0.033
	MPA		**0.229 (0.001)**		0.143 (0.241)		109.208 (41)	0.916	0.045 (0.035-0.055)	0.034
	LPA		0.103 (0.066)		0.137 (0.178)		105.910 (41)	0.918	0.044 (0.034-0.054)	0.034
	SED		**-0.157 (0.007)**		-0.142 (0.219)		107.410 (41)	0.913	0.044 (0.034-0.055)	0.034
**Balance skills**										
	TPA		0.077 (0.122)		0.028 (0.768)		43.067 (41)	0.998	0.008 (0.000-0.026)	0.017
	MVPA		**0.114 (0.010)**		0.083 (0.359)		39.779 (41)	1	0.000 (0.000-0.023)	0.017
	VPA		0.084 (0.077)		0.039 (0.655)		41.500 (41)	1	0.004 (0.000-0.024)	0.017
	MPA		**0.140 (0.001)**		0.139 (0.133)		37.902 (41)	1	0.000 (0.000-0.021)	0.017
	LPA		0.027 (0.602)		0.069 (0.335)		38.192 (41)	1	0.000 (0.000-0.021)	0.017
	SED		-0.072 (0.138)		-0.089 (0.281)		38.542 (41)	1	0.000 (0.000-0.022)	0.017

Estimate (p-value) for paths PA-FMS; FMS-PA. ME=Measurement error, TPA=total physical activity, MVPA=Moderate-to-vigorous physical activity, VPA=Vigorous physical activity, MPA=Moderate physical activity, LPA=Light physical activity, SED=Sedentary behavior. CFI=Comparative Fit Index, RMSEA=Root Mean Square Error of Approximation, SRMR=Standardized Root Mean Squared Residual. Models are adjusted for sex, age, BMI, parents' education level, FMS assessor, and group allocation. Significant associations are marked in bold. N=820.

Additional file 3

Table S3. Standardized coefficients for paths between physical activity and fitness measures from longitudinal models not corrected for measurement error, using observed fitness-scores (saturated model, i.e., no model fit indices).

	**Intensity**		**Contains PA ME**		
			**PA→FIT, est (p)**		**FIT→PA, est (p)**
**Handgrip strength**					
	TPA		**0.141 (0.008)**		0.096 (0.065)
	MVPA		**0.137 (0.012)**		0.078 (0.126)
	VPA		**0.139 (0.007)**		**0.116 (0.013)**
	MPA		**0.114 (0.032)**		0.012 (0.823)
	LPA		0.065 (0.119)		-0.069 (0.278)
	SED		**-0.106 (0.033)**		0.001 (0.989)
**Standing long jump**					
	TPA		**0.179 (<0.001)**		**0.164 (<0.001)**
	MVPA		**0.173 (<0.001)**		**0.114 (0.012)**
	VPA		**0.189 (<0.001)**		**0.174 (<0.001)**
	MPA		**0.126 (<0.001)**		0.009 (0.828)
	LPA		0.016 (0.629)		-0.051 (0.213)
	SED		**-0.089 (0.003)**		-0.032 (0.450)
**Motor fitness**					
	TPA		**-0.144 (<0.001)**		**-0.245 (<0.001)**
	MVPA		**-0.150 (<0.001)**		**-0.187 (0.005)**
	VPA		**-0.128 (<0.001)**		**-0.256 (<0.001)**
	MPA		**-0.158 (<0.001)**		-0.039 (0.585)
	LPA		**-0.097 (0.007)**		-0.014 (0.815)
	SED		**0.132 (<0.001)**		0.114 (0.077)

Estimate (p-value) for paths PA-FIT; FIT-PA. ME=Measurement error, TPA=total physical activity, MVPA=Moderate-to-vigorous physical activity, VPA=Vigorous physical activity, MPA=Moderate physical activity, LPA=Light physical activity, SED=Sedentary behavior. Models adjusted for sex, age, BMI, parents' education level, FMS assessor, and group allocation. Significant associations are marked in bold. N=820. Lower motor fitness estimates indicate better performance.

Additional file 4

Table S4. Standardized coefficients for paths between physical activity and fundamental motor skills, and goodness of fit indices for the longitudinal models in control group.

	**Intensity**						**Model fit**			
			**PA→FMS, est (p)**		**FMS→PA, est (p)**		**Chi-square (df)**	**CFI**	**RMSEA (90% CI)**	**SRMR**
**Locomotor skills**										
	TPA		**0.334 (0.003)**		**0.338 (0.053)**		45.474 (35)	0.984	0.026 (0.000-0.046)	0.031
	MVPA		**0.358 (0.001)**		0.331 (0.087)		45.379 (35)	0.986	0.026 (0.000-0.046)	0.032
	VPA		**0.317 (0.003)**		0.265 (0.191)		46.014 (35)	0.984	0.027 (0.000-0.046)	0.031
	MPA		**0.359 (0.001)**		0.311 (0.070)		47.757 (35)	0.982	0.029 (0.000-0.048)	0.033
	LPA		0.177 (0.097)		**0.249 (0.038)**		45.422 (35)	0.984	0.026 (0.000-0.046)	0.032
	SED		**-0.264 (0.015)**		**-0.371 (0.008)**		44.298 (35)	0.986	0.025 (0.000-0.025)	0.032
**Object control skills**										
	TPA		**0.296 (0.020)**		-0.392 (0.229)		102.394 (37)	0.862	0.064 (0.049-0.078)	0.045
	MVPA		**0.328 (0.002)**		-0.161 (0.621)		103.472 (37)	0.877	0.064 (0.050-0.079)	0.046
	VPA		**0.288 (0.009)**		-0.297 (0.327)		104.401 (37)	0.871	0.065 (0.050-0.079)	0.045
	MPA		**0.343 (0.002)**		0.059 (0.842)		102.484 (37)	0.878	0.064 (0.049-0.078)	0.047
	LPA		0.170 (0.082)		0.003 (0.983)		101.516 (37)	0.884	0.063 (0.049-0.078)	0.049
	SED		**-0.264 (0.011)**		0.037 (0.876)		100.059 (37)	0.883	0.062 (0.048-0.077)	0.047
**Balance skills**										
	TPA		0.120 (0.243)		-0.132 (0.429)		42.673 (37)	0.993	0.019 (0.000-0.040)	0.023
	MVPA		**0.154 (0.050)**		0.112 (0.462)		41.029 (37)	0.995	0.016 (0.000-0.038)	0.023
	VPA		0.139 (0.122)		0.034 (0.799)		42.228 (37)	0.994	0.018 (0.000-0.040)	0.023
	MPA		**0.152 (0.039)**		0.199 (0.280)		37.949 (37)	0.999	0.008 (0.000-0.035)	0.022
	LPA		-0.015 (0.872)		0.008 (0.939)		39.949 (37)	0.997	0.014 (0.000-0.037)	0.023
	SED		-0.064 (0.461)		-0.074 (0.542)		40.553 (37)	0.996	0.015 (0.000-0.038)	0.023

Estimate (p-value) for paths PA-FMS; FMS-PA. TPA=total physical activity, MVPA=Moderate-to-vigorous physical activity, VPA=Vigorous physical activity, MPA=Moderate physical activity, LPA=Light physical activity, SED=Sedentary behavior. CFI=Comparative Fit Index, RMSEA=Root Mean Square Error of Approximation, SRMR=Standardized Root Mean Squared Residual. Models are adjusted for sex, age, BMI, parents' education level, FMS assessor, and group allocation. Significant associations are marked in bold. N=437.

Additional file 5

Table S5. Standardized coefficients for paths between physical activity and fitness measures from longitudinal models in control group, using observed fitness-scores (saturated model, i.e., no model fit indices).

	**Intensity**		**PA→FIT, est (p)**		**FIT→PA, est (p)**
**Handgrip strength**					
	TPA		0.114 (0.324)		0.150 (0.310)
	MVPA		0.084 (0.420)		0.074 (0.479)
	VPA		0.097 (0.342)		0.160 (0.142)
	MPA		0.054 (0.575)		-0.040 (0.600)
	LPA		0.030 (0.696)		-0.102 (0.173)
	SED		-0.059 (0.525)		0.033 (0.706)
**Standing long jump**					
	TPA		**0.267 (<0.001)**		0.045 (0.735)
	MVPA		**0.233 (<0.001)**		-0.032 (0.715)
	VPA		**0.253 (<0.001)**		0.066 (0.543)
	MPA		**0.174 (<0.001)**		**-0.135 (0.025)**
	LPA		0.077 (0.112)		-0.074 (0.306)
	SED		**-0.151 (<0.001)**		0.037 (0.577)
**Motor fitness**					
	TPA		**-0.229 (<0.001)**		-0.242 (0.103)
	MVPA		**-0.225 (<0.001)**		-0.151 (0.214)
	VPA		**-0.204 (<0.001)**		**-0.260 (0.023)**
	MPA		**-0.216(<0.001)**		0.050 (0.682)
	LPA		**-0.115 (0.006)**		-0.014 (0.876)
	SED		**0.176 (<0.001)**		0.108 (0.305)

Estimate (p-value) for paths PA-FIT; FIT-PA. TPA=total physical activity, MVPA=Moderate-to-vigorous physical activity, VPA=Vigorous physical activity, MPA=Moderate physical activity, LPA=Light physical activity, SED=Sedentary behavior. Models adjusted for sex, age, BMI, parents' education level, FMS assessor, and group allocation. Significant associations are marked in bold. N=437. Lower motor fitness estimates indicate better performance.
